# Editorial: Tau Protein: Mechanisms From Health to Degeneration

**DOI:** 10.3389/fnmol.2021.743986

**Published:** 2021-11-01

**Authors:** Isabel Lastres-Becker

**Affiliations:** ^1^Department of Biochemistry, School of Medicine, Universidad Autónoma de Madrid (UAM), Madrid, Spain; ^2^Instituto de Investigación Sanitaria La Paz (IdiPaz), Instituto de Investigaciones Biomédicas “Alberto Sols” UAM-CSIC, Madrid, Spain; ^3^Centro de Investigación Biomédica en Red de Enfermedades Neurodegenerativas (CIBERNED), Madrid, Spain; ^4^School of Medicine, Instituto Teófilo Hernando, Universidad Autónoma de Madrid (UAM), Madrid, Spain

**Keywords:** TAU phosphorylation, TAU propagation, tauopathies, TAU treatment, TAU

In this Editorial, we are going to go through the essential points of the latest news published on the topic “TAU protein: mechanisms from health to degeneration.” In recent years, it has been shown that TAU protein is involved in multiple cellular mechanisms, which alterations are associated with neurodegenerative diseases called tauopathies. TAU is a neuronal microtubule-associated protein important for axonal transport which, under pathological conditions, forms aberrant assembly into insoluble aggregates. This led to synaptic dysfunction and neural cell death in a range of neurodegenerative disorders. Within this Editorial we will discover that the works presented are focus on three essential hallmarks of TAU: (1) TAU phosphorylation; (2) TAU oligomers and (3) TAU signaling (Figure 1). Altogether, finally, in this special issue, we will uncover several TAU-based therapies for these groups of diseases.

**Figure 1:**
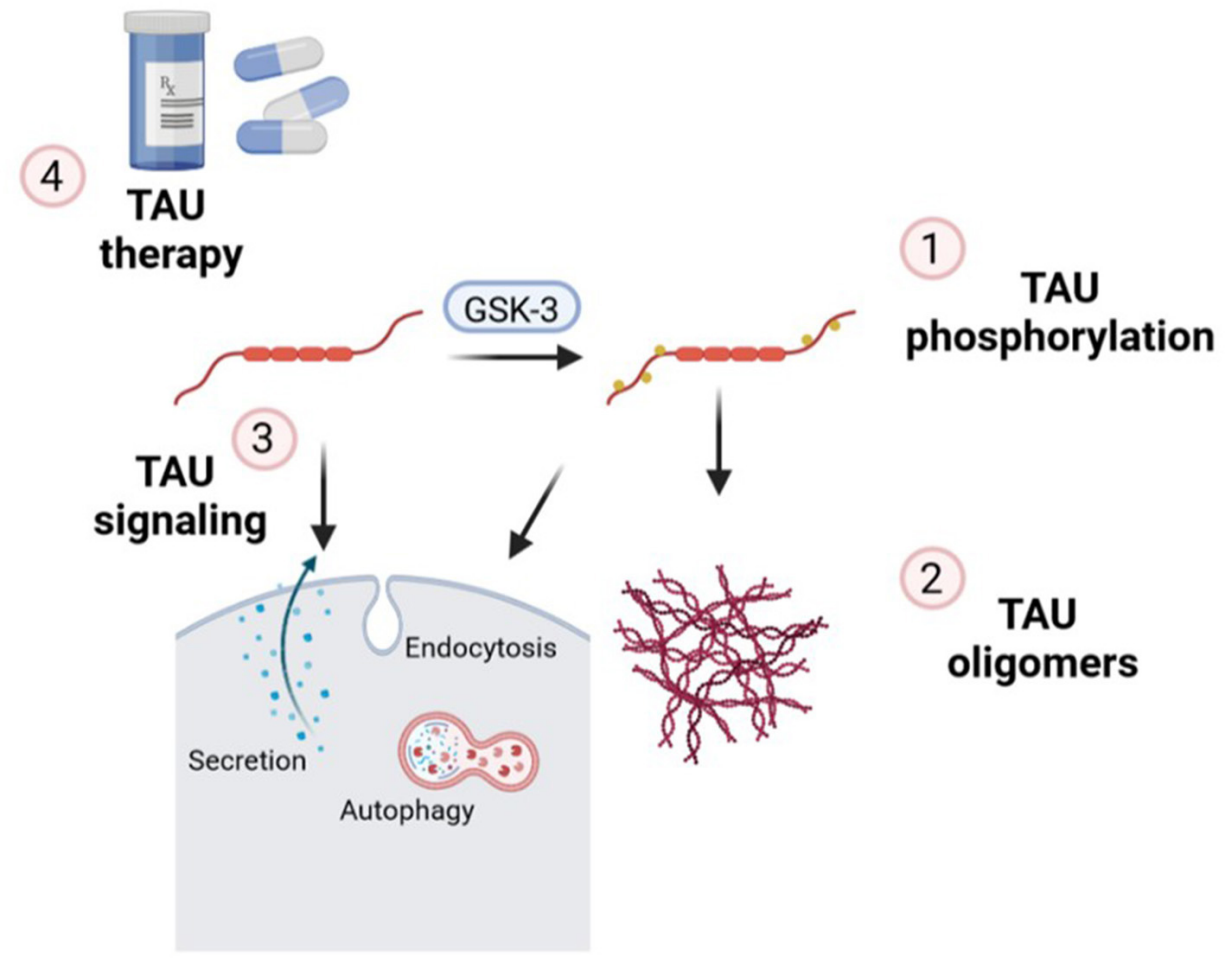
Essential points of the mechanisms of action of TAU in which new findings have been made on this topic and contextualized in this Editorial: (1) TAU phosphorylation; (2) formation of TAU oligomers; (3) implications of endocytosis, secretion, and autophagy in TAU signaling and (4) new insights related to TAU therapeutic strategies.

One of the most relevant aspects of the TAU protein is its phosphorylation. It has been described that this protein can be phosphorylated at more than 80 different sites due to the actions of multiple kinases (Morris et al.). Approximately 50 of these 80 sites are phosphorylated in normal brain and/ associated with Alzheimer's disease (AD) condition, 28 sites have been found phosphorylated only in AD brains, and 10 sites have not been fully characterized yet (Šimić et al., 2016). Furthermore, pseudophosphorylation (replacement of serine with glutamic acid or glutamate) of S199, S202, and T205 (at the AT8 antibody epitope) showed impact on TAU conformation that exposes a biologically active motif of the N-terminal of TAU to the action of protein phosphatase 1 (PP1)/glycogen synthase kinase 3 (GSK-3); (Jeganathan et al., 2006, 2008; Kanaan et al., 2011; Combs and Kanaan, 2017). This motif is the Phosphatase Activating Domain or PAD, which activates PP1/GSK-3 signaling pathway, which inhibits the anterograde fast axonal transport (FAT) mediated by kinesin. In this special issue, Morris et al. reported that differential phosphorylation of these three residues impact TAU conformation modulating its function as a signaling hub. T205 phosphorylation inhibited anterograde FAT through the exposure of N-terminal PAD and PP1/GSK-3β activation while S199 goes through the MAPK pathway involving JNK kinase. These results suggest that the phosphorylation of specific residues differentially impact TAU conformation leading to activation of different signaling pathways that influence differentially FAT. Concerning GSK-3, it has been described that the isoform GSK-3β is one of the main kinases involved in TAU phosphorylation although it is also implicated in neuronal proliferation and differentiation, neurite outgrowth and polarity as well as cytoskeleton stability (Rodríguez-Matellán et al.). While many efforts have been made to generate transgenic mice to evaluate the effects of overexpression of this enzyme in the central nervous system and its implication in the generation of AD, the adaptation phenomena that occur throughout the development process have prevented conclusive results. Therefore, within this specific Research Topic Rodríguez-Matellán et al. have generated a CamKIIα-tTA/GSK-3β mouse model to increase neuronal GSK-3β activity only during adulthood. The results demonstrated that this model reproduces the histopathological hallmarks of AD, like increased phosphorylated TAU, cell death and microgliosis. In line with the generation of novel transgenic models to study the contribution of specific phosphorylation sites of TAU protein, in this special issue, Keramidis et al. developed new Drosophila models to study the implication of S238, T245, and S262 of TAU protein in toxicity and learning. Their results point to phosphorylation of TAU at residue S262 as necessary to induce learning deficits and serve as “facilitatory gatekeeper” to S238 occupation, associated with TAU toxicity. Furthermore, phosphorylation at T245 behaves like a “suppressive gatekeeper” avoiding phosphorylation of different sites including S262 and therefore of S238. These data elucidate novel interactions among phospho sites of TAU protein implicated in toxicity and neuronal dysfunction. Initially, TAU hyperphosphorylation has always been thought to be one of the main causes of neuronal toxicity and death. But in this special issue, Siano et al. have shown that the Q336H mutation linked to Pick's disease lowers phospho TAU levels, stabilized TAU binding to tubulin and alters TAU conformation on microtubules. Furthermore, this mutation influences the cytoskeleton by increasing tubulin filament length and the number of branches. Although it appeared that this mutation had no apparent pathological effects, Siano et al. observed that this mutation induced the aggregation of TAU, forming intracellular inclusions. On the other hand, in AD, TAU hyperphosphorylation leads to TAU detachment from the microtubules and accumulation in intracellular neurofibrillary tangles. Dephosphorylation of AD hyperphosphorylated TAU by protein phosphatase 2A (PP2A) restored the microtubule polymerization activity of TAU (Wang et al., 1996). Hence, in this special topic, Wu et al. isolated oligomeric TAU (O-TAU) from AD brains and observed that was mainly composed of N-terminal truncated and C-terminal hyperphosphorylated TAU. Desphosphorylated O-TAU (Dp O-TAU) obtained by treatment with alkaline phosphatase showed decreased ability to capture free TAU and formed fewer aggregates. Furthermore, Dp O-TAU induced less accumulation of phosphorylated and total TAU in the insoluble fractions from cell lysates, indicating that dephosphorylation passivates the prion-like seeding activity of O-TAU (Wu et al.).

As it has been described, phosphorylated TAU forms oligomers, then fibrils and finally neurofibrillary tangles (NFTs; Hill et al.). The presence of a cysteine-291 residue is important for TAU aggregation (Soeda et al., 2015) and C291R mutation in the *MAPT* gene has been found in a patient diagnosed with corticobasal degeneration (CBD; Karikari et al.). In this special topic Karikari et al. showed that the C291R variant of TAU-K18 (a functional fragment with increased aggregation propensity) aggregated into a mix of (i) granular oligomers, (ii) amorphous aggregates of various sizes and shapes, and (iii) protofibrils, which appears to result from an *off-pathway process* related to fibril formation. Furthermore, they indicated that the C291R TAU-K18 aggregation mechanism seems to involve β-sheet-rich granular oligomers which reorganize to form distinctive protofibrillar structures, although they could not confirm the pathogenicity of C291R mutation and its link to CBD. Another factor that is linked to tauopathies is the deregulation of copper ions that might promote TAU hyperphosphorylation and the formation of TAU fibrils enriched in β-sheet, that can cause synaptic failure, neuronal death and cognitive deterioration, as in AD patients, as described by Zubčić et al. within this special topic. The accumulation of TAU aggregates and spread of misfolded TAU protein are the characteristics of tauopathies. Within this special issue of TAU protein, Merezhko et al. have reviewed the cell biology of TAU spreading in tauopathies, where TAU secretion could be due to three coexisting pathways: (1) translocation through the plasma membrane; (2) membranous organelles-based secretion; and (3) ectosomal shedding, although the involvement of different cell types cannot be ruled out. Related to tauopathies, like AD, the formation of paired helical filaments (PHF) of TAU can propagate in the brain in a prion-like fashion by recruiting endogenous TAU (Mudher et al., 2017). In this special issue, Houben et al. have analyzed the effects of intravenous injection of PHF-TAU obtained from AD brains in comparison to control brains into wild-type or the amyloid model 5XFAD mice. The results showed that a single intravenous injection of PHF-TAU proteins obtained from AD brains was enough to induce a long-term neuroinflammatory process in 5XFAD mice. These data indicated the modified TAU proteins present in blood could have a possible role in modulating the development of AD pathology in the brain of aged persons. This is because the permeability of the blood-brain barrier (BBB) increased with time (Michalicova et al.), indicating that the aged brain is more susceptible to crossing TAU proteins from plasma to the brain and potential seeding of TAU pathology (Houben et al.). Concerning tauopathies, the disruption of the BBB is driven by chronic neuroinflammation. Understanding the role of TAU in BBB structural and functional changes is essential to comprehend tauopathies. Within this special issue, Michalicova et al. have summarized the current knowledge relating BBB and TAU. Besides AD, other tauopathies like Argyrophilic Grain Disease (AGD; 4R tauopathy) and Primary Aged-Related Tauopathy (PART; 3R + 4R tauopathy) are also related to the seeding and spreading process. As described in this special edition, Ferrer et al. have inoculated sarkosyl-insoluble and sarkosyl-soluble fractions from “pure” AGD into the brain of wild-type mice and showed abnormal hyperphosphorylated TAU deposits in hippocampal neurons and grains and other brain areas. Similar results were obtained with sarkosyl-insoluble fractions obtained from PART. These results show the capacity for seeding and spreading of AGD-TAU and PART-TAU in the brain of wild-type mice and suggest that the characteristics of donor TAU and host TAU underlie common and specific characteristics of deposits both in human disease and corresponding experimentally induced murine models. To sustain TAU spreading from cell to cell it has been proposed that abnormal TAU uptake by endocytosis could be implicated (Wu et al., 2013; Rauch et al., 2020). Indeed, the endocytic machinery needs to be fine-tuned and is highly vulnerable. Therefore, within this special topic, Ando et al. have reviewed the implication of endocytosis dysfunction with TAU pathology in the context of AD.

Apart from the generation of the oligomeric forms of TAU and its spreading capacity, it is also interesting to decipher the mechanisms by which pathology occurs. In this special issue Hill et al. have summarized the possible electrophysiological approaches to investigate these aspects, like extracellular field activity at the circuit level, and patch-clamp at a single-cell level. One of the possible mechanisms associated with TAU toxicity could be due to alteration in the autophagic-endolysosomal network (AELN), which play an important role in TAU clearance (Jiang and Bhaskar). Defects in AELN have been found in several tauopathies, like AD, progressive supranuclear palsy (PSP) and frontotemporal dementia (FTD), where lysosomal dysfunction promoted the cleavage of TAU and its neurotoxicity. In this special edition, Jiang and Bhaskar have reviewed the implications of AELN in TAU toxicity at the neuronal level (where TAU fragments can lead to lysosomal dysfunction) and at the glial level (where microglia and astrocytes can uptake extracellular TAU). Therefore, AELN could be implicated in the degradation and/or transmission of TAU. Apart from the involvement of glia in AELN, the involvement of microglia and astrocytes in the control of plasticity at the brain level is also being recognized. Within this special topic, Koller and Chakrabarty have summarized the direct and indirect pathways in which astrocytes and microglia can modify brain plasticity in AD, with the development and progression of associated tauopathy. They highlight the role of non-neuronal cells in AD and their influence on neuroplasticity. Overall, there is evidence that indicates that TAU participates in multiple cellular processes at both the neuronal and glial levels through various mechanisms, involving the scaffolding and regulation of specific kinases and phosphatases, as reviewed in this special edition by Mueller et al. Within these features, TAU aggregation is an early event that starts to form ~15 years before clinical manifestations of the diseases. For this reason, special attention has been paid to its possible use as a diagnostic marker and monitoring of tauopathies. In this special issue Pizzarelli et al. have summarized the advances in biomedical methodology in TAU molecular imaging. TAU super-resolution imaging, PET and NEAR-infrared probes provide valuable information about TAU structure, interaction with proteins, and mechanisms of self-aggregation, although they have several limitations that need to be improved.

The study of all the mechanisms in which TAU protein is involved is essential to be able to establish new therapeutic strategies that are very necessary for patients with tauopathies since they currently do not have any treatment. As reviewed in this special topic by Soeda and Takashima, different pieces of evidence suggest several potential approaches to block TAU toxicity: (1) inhibit the different post-translational modifications that induce aggregation to TAU; (2) directly inhibit TAU aggregation; (3) inhibit the spread of TAU; (4) promote stabilization of microtubules. One of the most studied strategies is the modulation of TAU phosphorylation, either by hyperactivation of kinases or reduction of phosphatase activity. Protein phosphatase 2A (PP2A) accounts for ~71% of total phosphatase activity on TAU protein in the human brain (Liu et al., 2005), and has been found to be significantly in AD (Taleski and Sontag, 2018). In this special issue of TAU, Ahmed et al. demonstrated that chronic treatment with sodium selenate, an oxidized form of selenium that functions as a selective activator of PP2A, rescue synaptic plasticity deficiency and neurocognition of the hippocampus in THY-TAU22 mice. New alternative TAU-target therapies have emerged in the last years, like modulating liquid-liquid phase separation (LLPS) which can make a non-membrane-bound compartment in cells. As has been described before, a change of phase transition state by LLPS leads to the formation of a protein condensate referred to as liquid droplets. LLPS is an essential physiological and pathological event and some studies have revealed LLPS-mediated conversion of TAU protein to liquid droplets (Ambadipudi et al., 2017; Soeda and Takashima). Therefore, inhibition of TAU droplet formation could be a novel focal point of therapeutic strategy for tauopathies, as has been suggested in this special issue by Soeda and Takashima. But there are other pathologies where TAU is also implicated, although there are no considered to be typical tauopathies, like Huntington's disease (Baskota et al., 2019) or Parkinson's disease (Zhang et al., 2018). Finally, in this special topic, Mao et al. have demonstrated how the treatment with 6-amino-1-methyl-indazole (AMI), a novel small molecule that belongs to indazole derivates, inhibited TAU hyperphosphorylation and exerted neuroprotective effects in the 1-methyl-4-phenyl-1,2,3,6-tetrahydropyridine (MPTP) parkinsonism model. Consequently, the development of new therapies for tauopathies may also represent a repositioning of these therapies to other pathologies where TAU plays a relevant role.

This special edition highlights and details new mechanisms of the TAU protein and therapies for tauopathies. The data presented in this special edition indicate that TAU hyperphosphorylation is one of the main pathogenic events in tauopathies such as Alzheimer's disease (AD), Pick's disease (PD), corticobasal degeneration (CBD), Argyrophilic Grain Disease (AGD), and Primary Aged-Related Tauopathy (PART), which leads to the formation of oligomers and alterations involved in the seeding and spreading process of TAU protein. All this involves electrophysiological alterations, as well as impairment of the autophagic-endolysosomal network and the interactions with glial cells. Together, all these mechanisms are essential to establish new therapeutic targets that in the future may have an impact on the development and evolution of these tauopathies.

## Author Contributions

IL-B contributed to the drafting of the manuscript and figure.

## Funding

This work was supported by a Spanish Ministry of Economy and Competitiveness Grants Refs. PID2019-105600RB-I00 to IL-B; General Council for Research and Innovation of the Community of Madrid and European Structural Funds Ref. S2017/BMD-3813-ELA_Madrid to IL-B. Fundación Tatiana Pérez de Guzmán el Bueno P-043-FTPGB 2020 to IL-B. Fundela (2019/00325/001) to IL-B.

## Conflict of Interest

The author declares that the research was conducted in the absence of any commercial or financial relationships that could be construed as a potential conflict of interest.

## Publisher's Note

All claims expressed in this article are solely those of the authors and do not necessarily represent those of their affiliated organizations, or those of the publisher, the editors and the reviewers. Any product that may be evaluated in this article, or claim that may be made by its manufacturer, is not guaranteed or endorsed by the publisher.
